# Rectus Abdominis Muscle Endometriosis: A Unique Case Report with a Literature Review

**DOI:** 10.3390/cimb47010047

**Published:** 2025-01-13

**Authors:** Marijana Turčić, Koviljka Matušan Ilijaš, Koraljka Rajković Molek, Petra Valković Zujić

**Affiliations:** 1 Faculty of Biotehnology and Drug Research, University of Rijeka, Radmile Matejčić 2, 51000 Rijeka, Croatia; 2 Clinical Department of Pathology and Cytology, Clinical Hospital Centre Rijeka, Krešimirova 42, 51000 Rijeka, Croatia; koraljkarm@uniri.hr; 3 Department of Diagnostic and Interventional Radiology, Clinical Hospital Centre Rijeka, Krešimirova 42, 51000 Rijeka, Croatia; petra.valkovic.zujic@medri.uniri.hr

**Keywords:** abdominal wall endometriosis, case report, rectus abdominis muscle, incomplete surgical resection, unique clinical entity

## Abstract

Introduction and importance: Extrapelvic endometriosis, confined exclusively to the body of the rectus abdominis muscle, is a rare form of abdominal wall endometriosis. While its etiopathology remains unclear, it is often diagnosed in healthy women who present with atypical symptoms and localization unrelated to any incision site, or in the absence of a history of endometriosis or previous surgery. Presentation of the case: Here, we describe a unique case of intramuscular endometriosis of the rectus abdominis muscle in a healthy 39-year-old Caucasian woman. The condition was located away from any prior incisional scars and presented without typical symptoms or concurrent pelvic disease, making diagnostic imaging unclear. After partial surgical resection of the endometriotic foci, the diagnosis was confirmed histologically. Progestogen-based supportive medication was initiated to prevent the need for additional surgeries and to reduce the risk of recurrence. After 6 years of follow-up and continued progestogen treatment, the patient remains symptom-free and has shown no recurrence of the disease. Clinical discussion: Endometriosis of the rectus abdominis muscle exhibits specific characteristics in terms of localization, etiopathology, symptomatology, and diagnostic imaging, suggesting that it should be considered a distinct clinical entity. Conclusions: Although rare, primary endometriosis of the rectus abdominis muscle should be included in the differential diagnosis for women of childbearing age. Early diagnosis is essential to avoid delayed recognition, tissue damage, and to minimize the risk of recurrence or malignant transformation. Given the increasing frequency of gynecologic and laparoscopic surgeries worldwide, it is crucial to establish standardized reporting protocols, follow-up timelines, and imaging assessments during specific phases of the menstrual cycle. Standardization will help raise awareness of this disease, and further our understanding of its pathogenesis, risk factors, recurrence patterns, and potential for malignant transformation—factors that are still not fully understood.

## 1. Introduction

Endometriosis, defined as the presence of functional, ectopic endometrial tissue outside the uterus, is an invasive, benign, estrogen-dependent condition affecting up to 15% of women of reproductive age (approximately 285 million). Though it is responsible for infertility in 50% of cases and/or pain in up to 80%, the exact etiopathogenesis of endometriosis remains unclear [1,2,3,4,5]. Several theories have been proposed, including retrograde menstruation, mechanical transport, coelomic metaplasia, and vascular/lymphatic metastasis. The most widely accepted theory is Sampson’s transplantation theory, which suggests that endometrial tissue is reverse transferred during menstruation in women with an altered immune response [1,3].

Endometriosis is primarily localized in the pelvis, as ovarian or superficial peritoneal lesions, while deep infiltrating endometriosis (DIE) is characterized by lesions with more than 5 mm depth of invasion beneath the peritoneum or infiltration into the muscularis propria of hollow viscera. Deep dyspareunia (pain during sexual intercourse) is a common symptom of DIE, particularly in cases of rectovaginal septum endometriosis, a variant of DIE [6]. Interestingly, deep extrapelvic endometriosis, affecting approximately 6% of all women with endometriosis, can occur anywhere in the body. This makes it a significant diagnostic challenge, often leading to misdiagnosis and delayed treatment [1,4,7]. Though endometriosis is classified as a benign disease, it shares several similarities with cancer, such as tissue invasion, neoangiogenesis, cytological and architectural atypia, resistance to apoptosis, and spread to different organs [8].

Abdominal wall endometriosis (AWE) is one of the rarest forms of extrapelvic endometriosis, occurring in only 0.03% to 2% of cases. While it is commonly thought to result from iatrogenic implantation, typically following open or laparoscopic gynecological surgery, AWE has been reported in approximately 20% of cases without a history of abdominal surgery [2,9]. Concurrent pelvic endometriosis is present in up to 34% of cases; therefore, in women with umbilical AWE and no prior surgery, pelvic endometriosis should be highly suspected [1,2,3,5]. After cesarean section, the incidence of AWE is typically low, ranging from 0.03% to 0.4% [1]. Anatomically, the layers of the abdominal wall include the skin, subcutaneous fatty tissue, anterior rectus abdominis muscle (RAM) fascia, RAM, posterior RAM fascia, and the parietal peritoneum. Ectopic endometrial tissue can be found in any of these layers but is most commonly located in the skin or subcutaneous tissue near a previous surgical incision, sometimes extending to the fascia of the RAM [10]. However, rectus abdominis endometriosis (RAE)—a form of AWE where endometriotic lesions are confined to the body of the RAM—is extremely rare, with only a few cases reported to date. These cases typically affect women of reproductive age who have undergone prior gynecological or obstetric procedures, primarily cesarean sections [9]. It remains unclear whether the layer invaded by the lesion influences the likelihood of recurrence [11]. Moreover, due to its unusual localization and the absence of classic symptoms in about 50% of patients, the correct preoperative diagnosis is often difficult, delayed, and misdiagnosed, complicating the treatment approach [2,12].

## 2. Case Presentation

In accordance with the SCARE criteria [13], we present the case of a 39-year-old healthy Caucasian woman with a BMI of 19.0 kg/m^2^, who had no clinical or family history of endometriosis, no other comorbidities, and no history of medication use, allergies, or smoking. She visited her outpatient gynecologist in August 2018, reporting five months of increasing abdominal pain during exercise or physical strain and a painful, palpable, hard mass in the left infraumbilical region, which she had noticed a month earlier. She had undergone three cesarean sections, the first 10 years ago, the second 7 years ago, and the most recent 6 years ago, prior to the onset of her symptoms. No other gynecological issues were noted.

**Figure 1:**
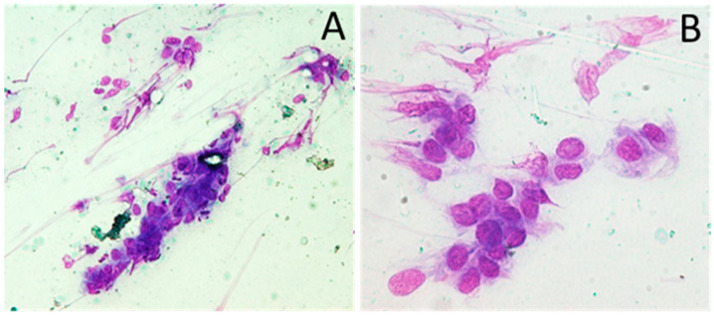
Cytologic examination of fine-needle aspiration. Fine-needle aspiration shows cohesive fragments consist of cells with crowding and overlapping of enlarged nuclei and indistinct cytoplasmic borders; May Grunwald-Giemsa stain, magnification ×100 (**A**), ×200 (**B**).

Physical examination revealed a 3 cm painful, hard, base-fixed mass in the left infraumbilical region, 10 cm above the cesarean scar, without skin lesions. Blood chemistry was normal, and the patient was referred to an abdominal surgeon with suspicion of muscle injury. Two weeks later, ultrasonography (US) conducted at a clinical hospital revealed a heterogeneous, mainly hypoechoic mass within the left RAM, measuring approximately 2 × 1.5 cm in diameter. Doppler sonography showed blood vessels within the lesion but no rapid blood flow. Due to the unclear etiology, US-guided fine-needle aspiration (FNA) was performed the same day. The results, obtained two days later, revealed scant cellularity with groups of atypical cells exhibiting crowding and overlapping of nuclei, moderate cytoplasm, and indistinct cytoplasmic borders (Figure 1).

**Figure 2:**
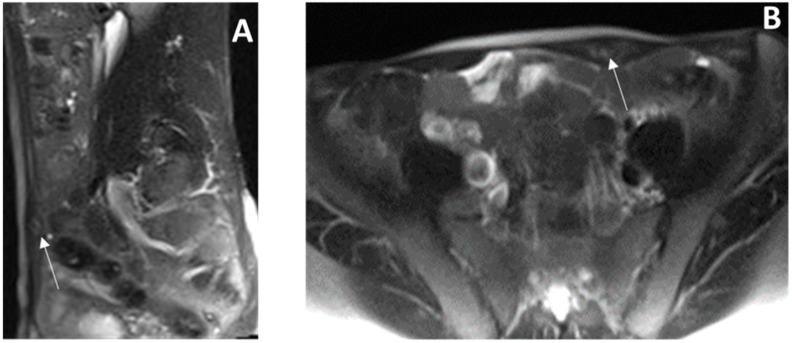
Magnetic resonance imaging (MRI) of abdomen and pelvis. Sagittal (**A**) and axial (**B**) HASTE sequence on magnetic resonance imaging (MRI) of abdomen and pelvis (like T2 weighted imaging with fat suppression) reveals enlarged left rectus abdominis muscle with ill-defined hyperintense lesion (arrow).

The findings were inconclusive for a definitive diagnosis, and a biopsy was recommended. Magnetic resonance imaging (MRI), performed a week later, confirmed a 2.1 × 0.9 cm focal lesion in the left RAM of unknown etiology (Figure 2). No other intra-abdominal lesions or associations with the cesarean scar were identified.

**Figure 3:**
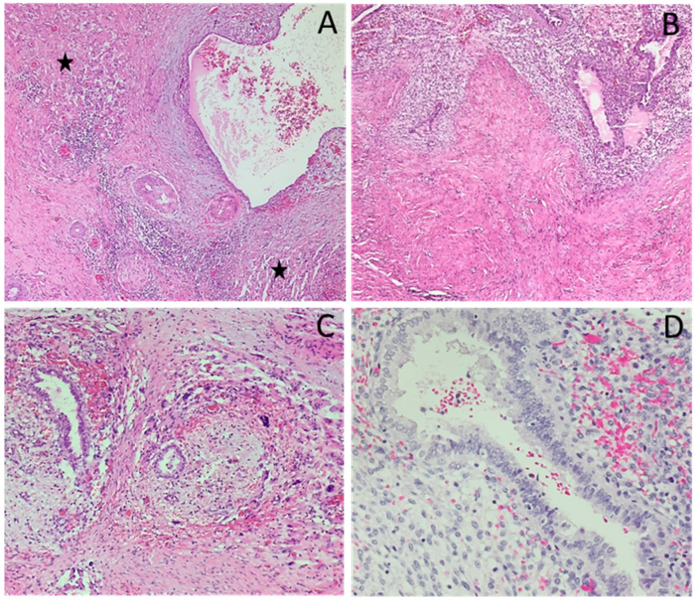
Pathohistological microscopic examination. Pathohistological analysis of the intramuscular node shows clusters of glands and the stroma characteristics of the endometrial mucosa. In the vicinity of the gland with a dilated lumen, the stroma of the endometrium is less noticeable, next to which the remnants of striated muscle tissue can be seen (asterisk) (**A**). Abundant connective tissue is seen around the glands and the clearly visible stroma of the endometrium, but the transverse striated musculature is not found (**B**). The focus of endometriosis with the microscopic foci of fresh bleeding is surrounded by connective tissue that permeates the fibers of the striated musculature with reparative changes (**C**). At high magnification, the epithelium of the gland is without atypia, showing mild proliferation, and there is scarce fresh bleeding in the surrounding stroma (**D**). Hematoxylin and eosin stain, magnification ×100 (**A**,**B**), ×200 (**C**), ×400 (**D**).

Five weeks after the patient’s first visit to her outpatient gynecologist, a plastic surgeon performed an excisional biopsy under local anesthesia, and the lesion was sent for pathohistological analysis. The insertion of a polypropylene mesh was not deemed necessary, and the patient was discharged the same day to home care. Microscopic examination revealed morphologically normal endometrial stroma and glands within the skeletal muscle tissue, with positive margins (Figure 3).

**Figure 4:**
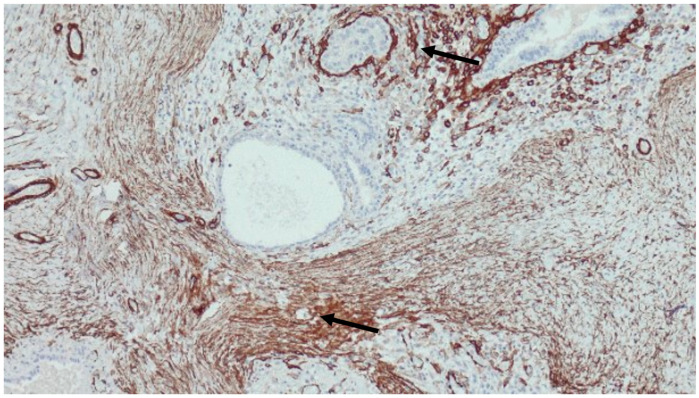
Alpha-smooth muscle actin (ASMA) immunohistochemical staining. Immunohistochemical staining for ASMA shows myoepithelial cells in the basal layer of the endometrial glands, but also within the stroma (arrows), magnification ×100.

Using immunohistochemistry, antibodies against alpha-smooth muscle actin (ASMA) revealed individual myofibroblasts in the stroma, migrating and subsequently joining together in bundles to form an architectural framework for fibrosis initiation in endometriotic foci (Figure 4).

**Figure 5:**
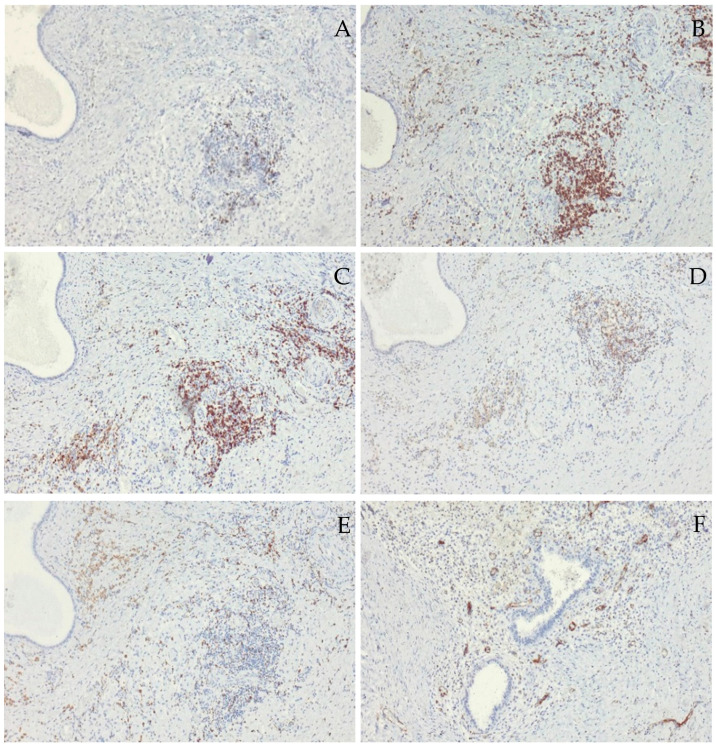
Immunohistochemical staining of inflammatory cells and blood vessels. Immunohistochemical staining using antibodies against specific cluster of differentiation (CD) molecules shows rare scattered B lymphocytes (**A**) and significantly more T lymphocytes (**B**) with a higher proportion of cytotoxic (**C**) than helper lymphocytes (**D**). Macrophages were present in a smaller percentage than T lymphocytes (**E**), while the stroma of endometriotic foci shows blood vessel proliferation (**F**); (**A**) CD20; (**B**) CD3; (**C**) CD8; (**D**) CD4; (**E**) CD68; (**F**) CD31; magnification ×100.

Furthermore, among non-epithelial, i.e., stromal cells in endometriotic foci, less than 1% of B lymphocytes (Figure 5A), up to 50% of T lymphocytes (Figure 5B–D), about 20% of macrophages (Figure 5E), and about 100 blood vessels per 1 mm^2^ were found (Figure 5F) using specific antibodies against cluster of differentiation (CD) molecules.

**Figure 6:**
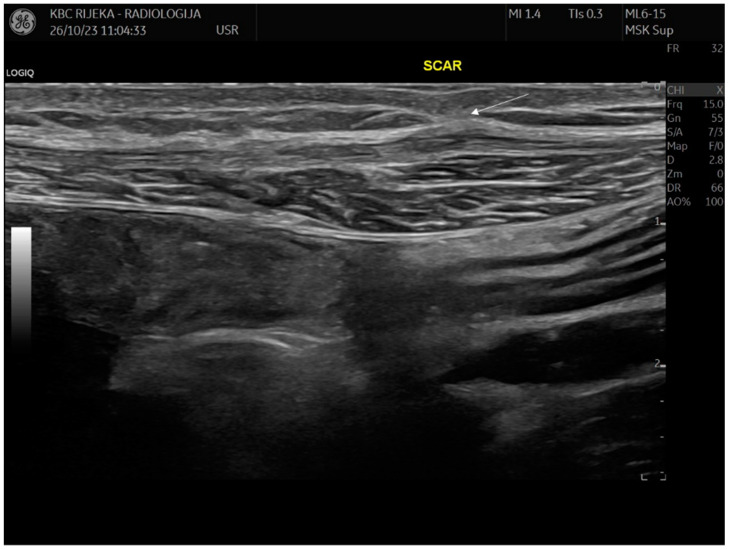
Anterior abdominal wall ultrasound examination. An ultrasound examination of the anterior abdominal wall with a linear 15 Hz probe 5 years after resection with minimal scarring (arrow) of the left rectal muscle without signs of recurrence.

The postoperative recovery was without complications, and the wound healed per primam. Further supportive treatment with progestogens (75 micrograms daily) was initiated to prevent additional surgery and the recurrence of endometriosis. After 6 years of follow-up, the patient remains symptom-free and has shown no recurrence of the disease (Figure 6).

This case, treated at our hospital, motivated us to explore this rare form of abdominal wall endometriosis more thoroughly. However, to date, the published literature on RAE has been scarce and inconsistent, primarily consisting of case reports or series. Seemingly, this is the first review on this particular topic.

## 3. Discussion

### 3.1. Etiopathogenesis

AWE presents as a solitary lesion in almost 95% of cases, typically located within or near the surgical scar, predominantly on the left side [14,15]. The formation of AWE, including in RAM, is considered iatrogenic, resulting from the mechanical transplantation of endometrial tissue during laparoscopic, gynecological, or obstetric procedures, most commonly cesarean sections. However, in 20% of women, AWE occurs without a history of surgery, arising spontaneously without any laparoscopic or surgical history [16].

Interestingly, after reviewing the literature published from 2000 to 30 June 2024, using PubMed and Google Scholar databases to identify relevant papers—including cases from our institution—we found only 49 reported cases of RAE. Of these, 51.02% were unrelated to the incisional site, while 85.71% of spontaneous AWE occurred within the RAM. The location of RAE was predominantly right sided (44.90%), while 22.45% of cases had an unknown location. Multifocal lesions within RAM or simultaneously within RAM and present subcutaneously were observed in 12.24% of cases. Moreover, considering the distribution of endometriosis in skeletal muscles, endometriosis originating in RAM was reported in 52.56% of cases [17]. RAE was predominantly (91.84%) diagnosed in healthy, fertile women between 16 and 48 years of age (mean age 35.45 years). Only one patient had a history of multiple sclerosis, one had infertility, one had hypothyroidism, and another had prior melanoma in situ.

Several theories regarding the etiology of extrapelvic endometriosis exist, but the pathogenesis remains controversial. The most widely accepted theory is Sampson’s retrograde menstruation theory, which suggests that endometriosis arises from the retrograde transfer of viable endometrial tissue during menstruation [1,3,18,19]. However, only 4 (8.16%) of the 49 women with RAE were diagnosed with concomitant pelvic endometriosis, indicating no direct association between RAE and other forms of endometriosis.

Since endometriosis is known to be associated with several diseases involving immune system dysfunction and a genetic component (e.g., multiple sclerosis, lupus, rheumatoid arthritis, Meniere’s disease, melanoma, non-Hodgkin’s lymphoma, ovarian and breast cancer), it is possible that immune dysregulation could trigger the development of endometriosis [20]. In line with our case, some studies have shown an increase in the proportion of B lymphocytes in endometriosis, while others reported a decreased proportion. Additionally, there are studies suggesting an increase in T regulatory lymphocytes (Tregs), which may cause an imbalance between T helper cell types (Th1 and Th2) [21,22].

Macrophages also play a dual role; they can both stimulate and inhibit the development of endometriosis, depending on their origin. Studies indicate that macrophages originating from the endometrium promote endometriosis, while those derived from monocytes have an anti-endometriotic effect [23]. Thus, immune cells play a significant role in immunomodulation and may either promote or inhibit the development of endometriosis depending on their function. However, the existing literature does not provide definitive conclusions regarding their number or role in the development of endometriosis, and further research is needed.

Furthermore, studies have shown that endometriosis is associated with a higher density of blood vessels compared to a normal endometrium, and deep endometriosis is characterized by an even higher vascular density [24,25,26]. In our case, about 100 blood vessels per 1 mm^2^ were observed, which is consistent with the findings of other studies. Recent studies on women with pelvic endometriosis have also reported endometriosis-associated eutopic endometrial aberrations, such as a higher number of basalis-like cells in the stratum functionalis of the eutopic endometrium, which have the potential to differentiate into endometrial stem cells crucial for ectopic lesion formation [27,28,29].

Additionally, the metastatic theory of vascular or lymphatic spread posits that endometrial stem cells may passively infiltrate the angiolymphatic vessels during menstruation, entering circulation and locating in an environment suitable for seeding. This could explain the occurrence of extrapelvic endometriosis, including RAM endometriosis, likely due to immune dysfunction [3,9,30,31,32]. The coelomic metaplasia theory suggests that the original coelomic epithelium may undergo metaplasia, transforming into endometrial glands and stroma [33]. Another possibility is that metaplastic myofibroblasts arise from endometriotic stromal cells or neighboring mesenchymal cells. Myofibroblasts have been identified in all three types of endometriotic lesions. Moreover, it has been reported that the endometriotic stromal cells in RAE strongly express ASMA, a marker of myofibroblasts [34,35,36,37].

Indeed, the contractility of myofibroblasts may contribute to the chronic pain and pain related to physical strain often reported in women with RAE. It is important to consider the possibility that different etiologies may exist for the various origins of extrapelvic endometriosis.

### 3.2. Symptomatology and Diagnosis

AWE usually presents as a localized, painful, and palpable mass near a previous incision scar, accompanied by cyclic abdominal pain that intensifies during menstruation [30]. A history of prior abdominal or laparoscopic surgery is common (77%), with the time interval between the initial surgery and the onset of symptoms ranging from 1 to 24 years (mean 4.8 years) [9]. However, these symptoms occur in only 50% of cases [38]. In some instances, pain may be absent, or the pain may be chronic or acute, unrelated to the menstrual cycle. Additionally, the lesion may be impalpable, or only superficial skin changes above the lesion may be present [14]. Interestingly, non-cyclic pain is more frequently associated with endometriotic lesions located within the RAM (40.82%). This pain in women with RAE is often related to physical strain (8.16%), likely due to endometriotic foci limited solely to the RAM. In 24.49% of cases, the mass was impalpable, while 12.24% of women with RAM reported no pain.

Diagnosing RAE, especially when it is unrelated to the incision site and presenting with nonspecific symptoms, is challenging. Differential diagnoses include abscess, granuloma, hematoma, lipoma, neuroma, lymphadenopathy, lymphoma, inguinal or postoperative incisional hernia, primary or metastatic cancer, sarcoma, neurofibroma, and desmoid tumor [1]. In fact, RAE was considered as a differential diagnosis in only 38.78% of the reviewed cases.

Non-invasive diagnostic tests generally have a low sensitivity for detecting endometriosis. There are currently no specific laboratory tests for endometriosis. Serum levels of cancer antigen 125 (Ca 125), a biomarker for ovarian cancer, are not specific to endometriosis and are often only slightly elevated in women with the condition [39]. While Ca 125 may be useful in monitoring treatment or diagnosing moderate to severe endometriosis, it is not reliable for distinguishing between endometriosis-associated ovarian cancers and ovarian endometriomas [40]. As per international guidelines, routine testing of Ca 125 is not recommended due to its low sensitivity [41,42]. Other biomarkers, such as C-reactive protein (CRP), Ca 19-9, folistatin, activin A, and anti-Müllerian hormone, have been reported to show alterations, but none have proven to be substantially specific for endometriosis [41,43,44,45,46,47]. Nevertheless, a combination of biomarkers should be considered when laboratory testing is necessary.

Among women with RAE, most cases involved routine blood tests that were within normal range. Serum Ca 125 levels were measured in 10.20% of cases, with slight elevations noted in one case of reported recurrence of RAE with double lesion sites, alongside elevated Ca 19-9 levels [12]. However, since endometriotic lesions in RAM are solitary in most cases (87.76%) and often occur in women without pelvic endometriosis, altered serum biomarkers are rarely expected. These biomarkers are, however, often monitored in follow-up programs for women after surgical excision of AWE.

Despite significant advancements in imaging diagnostics, AWE imaging findings are typically nonspecific, making early preoperative diagnosis difficult, especially in the absence of clinical symptoms and signs [2,9,38]. On ultrasound, endometriotic foci appear as solid, iso-, hyper-, or hypoechoic masses, or as echogenic cystic or polycystic forms with irregular, often needle-like margins infiltrating surrounding tissue, with peripheral vascularization present in 61.5% of cases. A hyperechoic ring around the lesion is frequently seen, likely due to an inflammatory response in adjacent tissue. CT imaging typically shows a solid, well-demarcated, homogeneous, and hypervascular mass, while MRI features may vary, with areas of low and high signal intensity on T1- and T2-weighted images, suggesting old hemorrhage or fibrosis. The infiltration of muscle fascia and contrast enhancement may also be observed [12,14,38,48,49,50]. A high T1 signal often indicates intralesional hemorrhage, while a low T2 signal around the lesion is highly specific for endometriosis. MRI can significantly improve AWE diagnosis due to its ability to distinguish hemorrhagic signals in endometriotic lesions [51].

In RAE reports, ultrasound (US) imaging was the most commonly used technique (61.22%), followed by MRI (48.99%) due to its high spatial resolution, which is important for detecting small lesions, and CT imaging (30.61%). Only one report mentioned performing gastrointestinal endoscopy due to iron deficiency anemia secondary to menorrhagia, and another involved a double contrast barium enema due to bowel symptoms in patients with multiple sclerosis [18,52]. Imaging findings in RAE cases, however, are inconsistently reported.

In the RAE cases examined, US features primarily showed a hypoechoic, solid mass in the RAM, which was well vascularized in 63.33% of cases. Only 16.67% of cases exhibited peripheral vascularization, and no hyperechoic surrogate ring was noted. On CT scans, RAE appeared as a predominantly isointense, solid mass with mild or severe contrast enhancement. MRI often showed a heterogeneous, hyperintense mass with enhancement after contrast administration. Occasionally, areas indicative of hemorrhage (30.43%), cystic structures (8.70%), and irregular margins were observed. Although the echo pattern may not always align with menstruation, imaging findings in both AWE and RAE are influenced by the menstrual cycle, bleeding levels, glandular and stromal tissue amounts, and the surrounding inflammatory and fibrotic response [1,3,53]. This suggests that imaging of AWE and RAE should be performed in accordance with the menstrual cycle phase to improve diagnostic accuracy and address case heterogeneity. This is consistent with recent findings related to deep infiltrating endometriosis (DIE) [54]. Nonetheless, imaging techniques, particularly MRI, are crucial for determining the location, depth, extent, and size of endometriotic lesions, even though they remain nonspecific. The accuracy of diagnosis depends largely on the lesion’s location, the patient’s surgical history, and the presence of concomitant pelvic endometriosis.

Ultrasound-guided fine-needle aspiration (FNA) is a simple and useful procedure for ruling out malignancy, but it is inconclusive in up to 75% of AWE cases and is associated with an increased risk of recurrence. Consequently, FNA is generally not recommended [1,2,55]. Two of three criteria must be met for a positive FNA diagnosis: the presence of endometrial stroma, endometrial glands, or hemosiderin histiocytes [47,56]. In RAE reports, FNA was performed in 14.29% of cases, with 57.14% being inconclusive. In contrast, US- or CT-guided biopsy was performed in 16.33% of cases, yielding positive results in 87.5% of cases.

### 3.3. Treatment

Pre- and postoperative hormone therapy, including gonadotropin-releasing hormone agonists, progesterone, danazol, or combined oral contraceptives, may alleviate symptoms in patients with endometriosis but does not provide a cure, as symptoms often recur after discontinuation of the medication. However, up to 33% of patients experience intolerable side effects or progesterone resistance [57,58]. Clinical trials are ongoing to investigate selective progesterone and estrogen receptor modulators, as well as aromatase inhibitors. According to the ESHRE guidelines, progestins are now considered the first choice for treating endometriosis due to their effectiveness and lower prevalence of side effects, particularly in delaying surgery or preventing disease recurrence after surgery [57,58,59].

The recurrence rate for AWE is 4.3%, often linked to unclear surgical margins [16]. If the lesion recurs or rapidly increases in size, malignant transformation should be suspected [60,61]. The malignancy risk in endometriosis is approximately 1%, with 20% of these malignancies being extrapelvic [62,63]. The prevalence of malignant AWE in previous scars is extremely rare (1%), with a latency period between 4 and 41 years [62,63,64,65]. Notably, malignant AWE is almost always associated with prior gynecological or obstetric procedures (98.6%), particularly cesarean sections (89%), and arises exclusively within or near previous surgical scars, often involving RAM and potentially extending into the peritoneal cavity [66,67,68,69]. Additional risk factors for malignant AWE include a positive history of endometriosis, hyperestrogenism, postmenopausal age, and lesion size greater than 9 cm [68,70,71]. Reactive oxidative stress, aberrant DNA methylation, genetic anomalies like p53 mutations, the loss of heterozygosity on chromosome 4 or AR1D1A, PTEN mutations, and exposure to carcinogens such as dioxin are also implicated in malignant transformation [69,72,73,74].

The most common histologic types of malignant extrapelvic endometriosis are endometrioid adenocarcinomas (69.1%) and sarcomas (25%), with clear cell carcinoma observed in only 4.5% of cases. Interestingly, AWE malignancies are predominantly clear cell carcinomas (66%), followed by endometrioid carcinoma (24%). The five-year survival rate for malignant AWE ranges from 40% to 80%, with better outcomes for endometrioid adenocarcinomas [64,66,69,70].

Since AWE malignancies arise exclusively within or near previous surgical scars, and RAE is often unrelated to incisional sites or arises spontaneously without prior surgery, it is possible that RAE does not carry the same malignancy risk. Unfortunately, the origin of malignant AWE is unclear in most reported cases, and it cannot be conclusively confirmed whether malignant AWE originates from RAM.

There are promising non-surgical treatments for AWE, such as cryoablation, ultrasound-guided intralesional ethanol injection, radiofrequency ablation, ultrasound-guided high-intensity focused ultrasound, and microwave ablation. However, extensive local excision with 5-10 mm surrounding margins remains the treatment of choice, although great care must be taken to avoid rupture of the lesion and reimplantation of microscopic endometrial fragments, which could lead to recurrence [14,17,75,76,77,78,79,80]. In cases where significant fascial defects occur after excision, parietal reconstruction with polypropylene mesh may be required [1,2].

Among women with RAE, surgical excision with pathohistological confirmation was performed in 95.92% of cases, with 89.80% of these cases being definitively treated by surgery. Only one successful case of ultrasound-guided intralesional ethanol injection treatment was reported [76].

Given that surgical incisions themselves pose a risk for AWE recurrence, complete surgical excision may lead to large abdominal wall defects, wound complications, and poor cosmetic outcomes. In some cases, surgical excision may be contraindicated or impossible, making percutaneous non-surgical treatments a preferred option. Non-surgical interventions have been shown to be as effective and safe as excision surgery, with shorter hospitalization times [79]. A multidisciplinary approach involving different healthcare providers is crucial for determining the most appropriate treatment plan, optimizing patient care, and minimizing the risk of recurrence.

Hormone therapy is often recommended before surgery to reduce the size of endometriotic lesions, as well as in cases of suspected incomplete resection [1,17,57]. After complete surgical resection, the recurrence rate is very low, making postoperative hormone therapy unnecessary [2]. In the literature, adjuvant hormonal treatment with oral contraceptives or norethisterone was prescribed for 90 days after complete surgical excision in two reported cases (4.35%) to prevent recurrence [75,81]. In our case, progesterone treatment was prescribed after incomplete resection of the endometriotic lesion, with no recurrence during a 5-year follow-up period. One patient with RAE was treated solely with hormonal therapy (leuprolide acetate) for 12 months [76], while another received hormonal therapy for 8 years before undergoing surgical excision for RAE recurrence [12]. Remarkably, this was the only reported case of RAE recurrence, which occurred 13 years after the initial AWE excision. The mean follow-up time in the literature was 9.45 months (ranging from 3 to 70 months), with no further recurrences reported. However, the literature on RAE is primarily based on case reports from various specialties, often lacking consistent or comprehensive patient data and long-term follow-up, making conclusions about recurrence and malignant transformation speculative.

Given the presence of metaplastic myofibroblasts in various forms of endometriosis, including AWE, myofibroblasts have been proposed as potential therapeutic targets. In vitro studies suggest that inhibiting ASMA could reduce myofibroblast contractility, potentially decreasing tissue micro-disruption and the likelihood of new endometrial fragments being shed and implanted [35,82,83,84]. However, this type of treatment has not yet been reported in clinical practice.

## 4. Conclusions

Endometriotic lesions confined solely within RAM represent a rare and poorly understood form of AWE. The delayed diagnosis of this condition, largely due to its rarity, inconclusive imaging findings, a broad differential diagnosis, and controversial pathogenesis, poses a significant challenge for healthcare professionals. This is especially true for RAE, which often presents with atypical symptoms and occurs in locations unrelated to previous incisions or in women with no history of endometriosis or prior surgeries.

This report is the first comprehensive review of this unique form of AWE, and our findings represent a crucial step towards better understanding of RAE. We aim to define it as a distinct clinical entity. To establish an evidence-based approach for its diagnosis and treatment, it is imperative to develop standardized protocols for reporting and long-term follow-up of RAE. Additionally, conducting imaging during specific phases of the menstrual cycle will likely improve diagnostic accuracy.

Standardizing diagnostic and reporting practices will not only enhance our understanding of RAE’s pathogenesis but also help identify key risk factors, recurrence rates, and the potential for malignant transformation. Such efforts are particularly important given the increasing global prevalence of gynecological and laparoscopic surgeries.

Immune cells, such as lymphocytes and monocytes/macrophages, play a significant role in immunomodulation and can either promote or inhibit the development of endometriosis, depending on their function. However, the current literature does not provide reliable conclusions regarding the exact number of these immune cells or their specific role in the pathogenesis of endometriosis. Understanding these factors would be crucial in advancing our knowledge of the disease. Therefore, further research is essential to draw definitive conclusions about the immune response in endometriosis.

## Data Availability

Data are contained within the article.
